# A new subspecies of *Seseli gummiferum* (Apiaceae) from Ilgaz Mountain National Park, northern Turkey

**DOI:** 10.3897/phytokeys.56.5755

**Published:** 2015-10-06

**Authors:** Özlem Çetin, Meryem Öztürk Şeker, Ahmet Duran

**Affiliations:** 1 Department of Biotechnology, Selçuk University, Selçuklu, Konya, Turkey; 2 Department of Biology, Selçuk University, Konya, Turkey

**Keywords:** New taxon, *Seseli gummiferum*, Turkey, Umbelliferae

## Abstract

A new subspecies Seseli gummiferum Pall. ex Sm. subsp. ilgazense A.Duran, Ö.Çetin & M.Öztürk, **subsp. nov.** (Apiaceae) is described from Kastamonu province, Turkey. It was collected from the open *Pinus sylvestris* L. and *Abies nordmanniana* (Steven) É.Spach. mixed forest in the northern Anatolian region. An endemic apparently confined to the Ilgaz Mountain National Park, the new taxon is closely related to Seseli gummiferum subsp. gummiferum. Diagnostic morphological characters for closely similar taxa are discussed, and a key to the subspecies of *Seseli gummiferum* is presented. ITS (Internal Transcribed Spacer) region of the nuclear ribozomal DNA of closely related *Seseli* L. taxa and *Pimpinella* is used to constract phylogenetic tree by using BioEdit and Seaview Programme.

## Introduction

The Apiaceae comprise approximately 450 genera and 3700 species worldwide (Pimenov and Leonov 1993). However, the distribution of species among the genera vary, with almost half of the genera monotypic and 26% consisting of only two or three species. Sixty percent of the species in the family assign to just a few genera, which genera encompass over 20 species have been noted as polyphyletic (Spalik et al. 2004). Asian countries with the greatest biodiversity for the Apiaceae include China, Asian Turkey, Iran, Asian Russia, and Kazakhstan, with the most species given for the Chinese flora (677 species in 108 genera) (Pimenov and Leonov 2004). Turkey, with a considerably smaller geographic area, is second only to China in its diversity for the Apiaceae, with 450 species in 109 genera. There are four endemic genera in Turkey, with 140 species among 42 genera. This suggests that the Asiatic region in Turkey has the highest known species-level diversity for the Apiaceae in Asia, if not in the world (Pimenov and Leonov 2004).

*Seseli* L. is one of the largest genera in the Apiaceae with 125 to 140 taxa ascribed to the genus worldwide. Represented by both intraspecific and interspecific diversity, *Seseli* is distributed in Europe, Asia, Africa, North America and Australia (Pimenov and Leonov 2004). The first revision of *Seseli* in Turkey was made by Hedge and Lamond (1972), who recognized 10 infrageneric taxa. Seseli gummiferum subsp. gummiferum was reported as a new record from Turkey (Duman 2000). After that *Seseli ramosissimum* replaced with *Seseli hartvigiii* by Parolly (Parolly and Nordt 2001). Latest revision of *Seseli* in Turkey was made by Doğan Güner and Duman (2013). So the total number of these taxa is 13 now.

## Material and methods

In 2008, during a field trip in the Black Sea region of Turkey, an unusual specimen of *Seseli* was collected by the authors. Study of the descriptions in Hedge and Lamond (1972), Davis et al. (1988), Duman (2000), Ball (1968), Rechinger (1987), Parolly and Nordt (2001), Shishkin (1950), Özhatay et al. (2009), Doğan Güner and Duman (2013) as well as comparison with herbarium material in GAZI, HUB, KNYA and ANK revealed that the specimens indeed represented a new taxon. In particular, the new taxon was compared with the closely similar taxa Seseli gummiferum Pall. ex Sm. subsp. gummiferum and *Seseli corymbosyum* Boiss. & Heldr. In the morphological description below, each numerical value is the average of ten measurements from different specimens. The abbreviations of the authors of plant names were checked from Brummitt and Powell (1992).

**DNA isolation**: Total DNA was obtained from 50–75 mg leaf and fruit from six different individuals. DNAs are isolated with CTAB method and after concentrations were determined by Nanodrop. Sample DNAs were diluted 25 ng/µl. Stok DNAs were kept at -86 °C.

**ITS amplifications**: ITS region of studied taxa were amplified using ITS4 (5' TCC TCC GCT TAT TGA TAT GC 3') and ITS5 (5' GGA AGG AGA AGT CGT AAC AAG 3') primers. PCR condition is 95 °C for 5 min initial denaturation, 35 cycles of 94 °C for 30 s denaturation, 50 °C for 30 s anneling, and 72 °C for 1 min extension, 72 °C for 10 min final extension.

**Data collection and cluster of phylogenetic analysis**: PCR products were visualised by agarose jel. The amplified fragments were sequenced using the same primers used for amplification. ITS sequences of the taxa were aligned via Bioedit and were used to construct phylogenetic trees by using Seaview.

## Taxonomic treatment

***Seseli gummiferum gummiferum* Pall. ex Sm., Exot. Bot. [Smith] ii, 121 (1807).**

Replaced synonym: *Bubon rigidus* Spreng., Syst. Veg. (ed. 16) [Sprengel] 1: 900 1825 [1824], nom. illeg., non Bubon rigidus (Waldst. & Kit.) Spreng. Pl. Min. Cogn. Pug. 2: 53. 1815. Type: [London] Cult. in Oxford Bot. Garden and Hort. Lady Hume.

### *Seseli gummiferum crithmifolium* (Boiss.) P.H.Davis, Notes Roy Bot. Gard. Edinb. 21: 120 (1953).

Basionym: *Seseli crithmifolium* Boiss., Fl. Orient. [Boissier] 2: 962 (1872). Type: Greece, Insulae maris Aegei, *Tournefort* 324 (holotype P, photo!, E!).

#### Seseli gummiferum ilgazense

Taxon classificationPlantaeApialesApiaceae

A.Duran, Ö.Çetin & M.Öztürk subsp. nov.

urn:lsid:ipni.org:names:77150275-1

**Figure 1.**
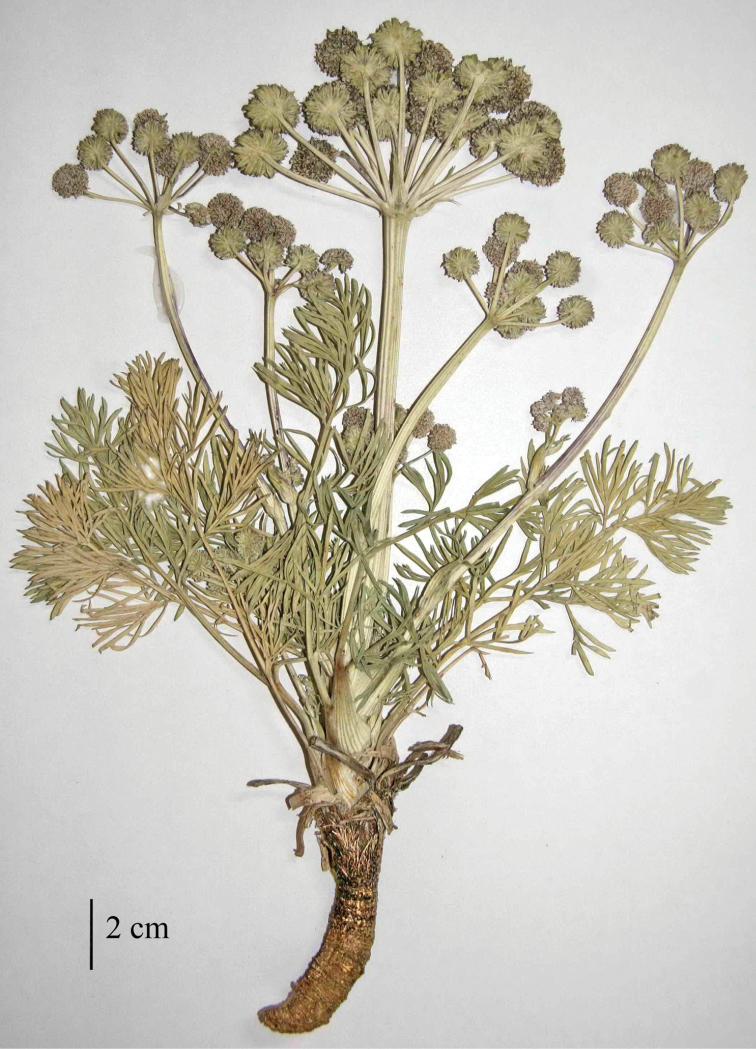
Seseli gummiferum subsp. ilgazense A.Duran, Ö.Çetin & M.Öztürk, subsp. nov.

**Figure 2.**
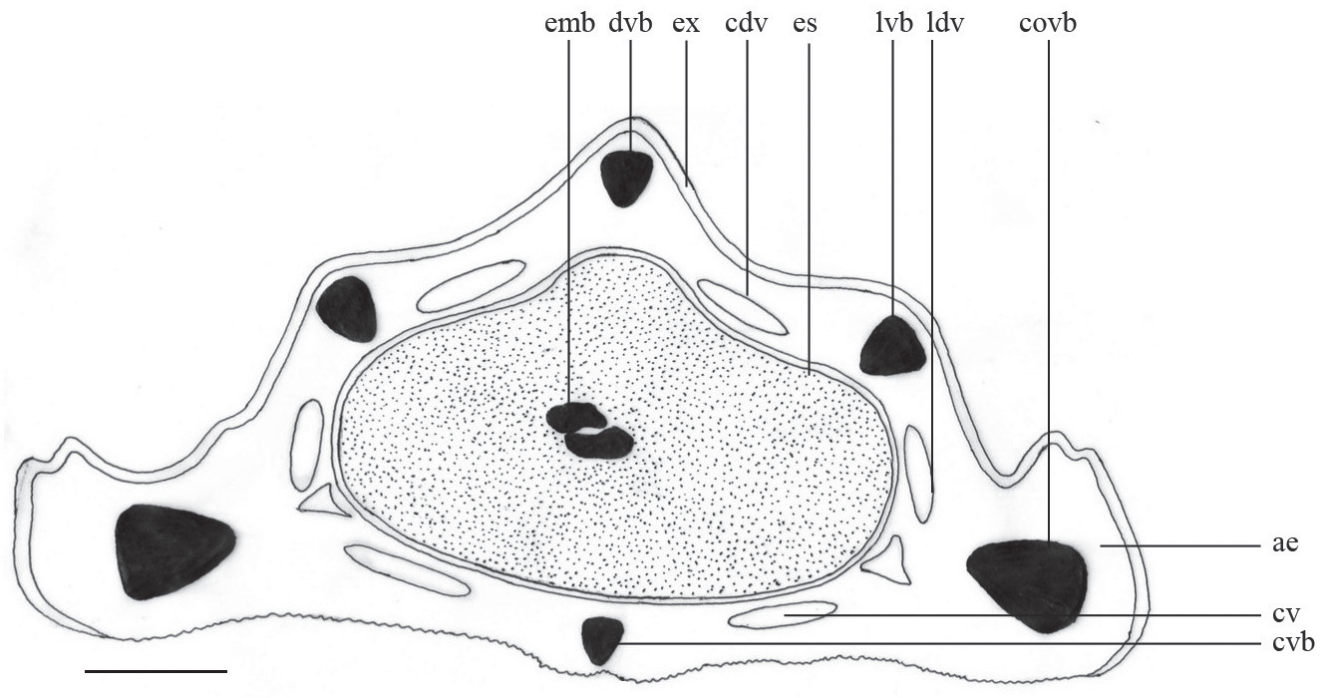
Cross section of fruit: emb = embriyo; dvb = dorsal vascular bundle; ex = exocarp; cdv = central dorsal vittae; es = endosperm; lvb = lateral vascular bundle; ldv = lateral dorsal vittae; ae = aerenchyma; cv = commissural vittae; cvb = carpophore vascular bundle; covb = commissural vascular bundle. Scale bar = 0.2 mm.

**Figure 3.**
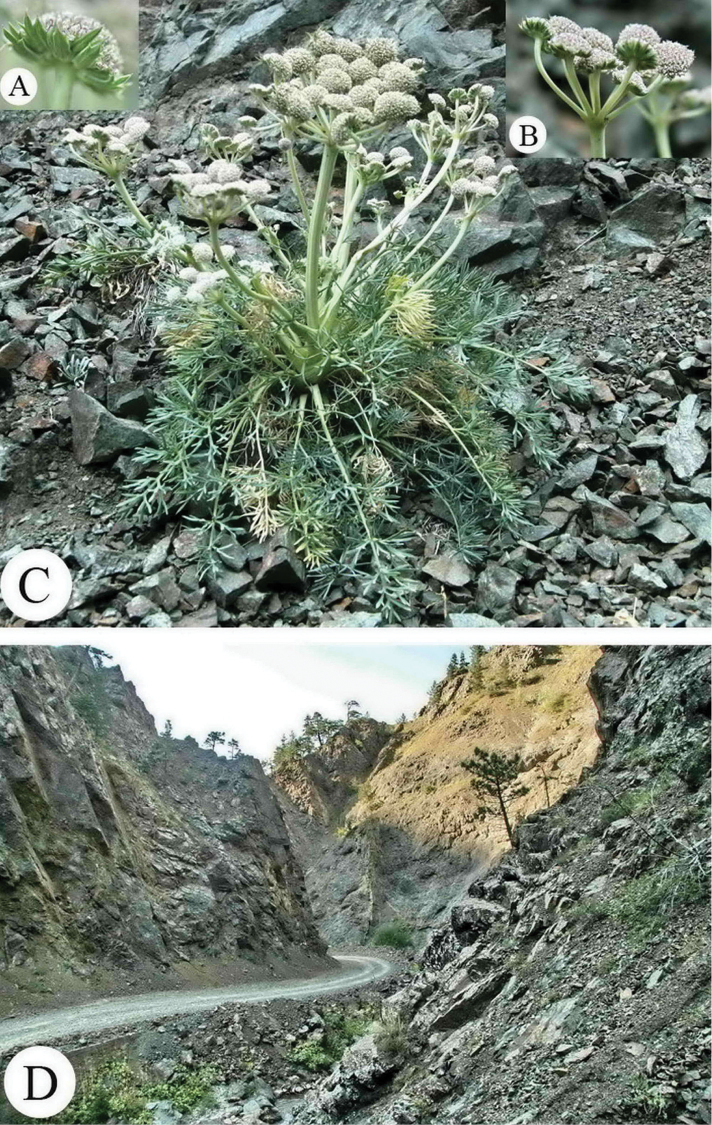
Seseli gummiferum subsp. ilgazense. **A** trifid and entire bracteoles **B** lateral umbel **C** general aspects **D** habitat and general view of type locality. Photo by A.Duran.

**Figure 4.**
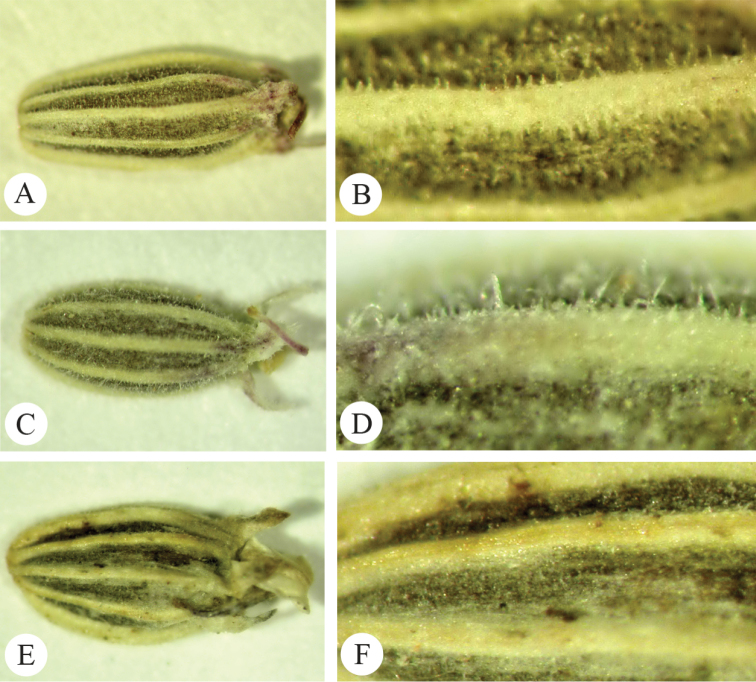
Comparison of *Seseli gummiferum* fruits. *Seseli corymbosum* fruits. **A** general view **B** surface details. *Seseli corymbosum*: **C** general view **D** surface details. Seseli gummiferum subsp. ilgazense: **E** general view **F** surface details.

**Figure 5.**
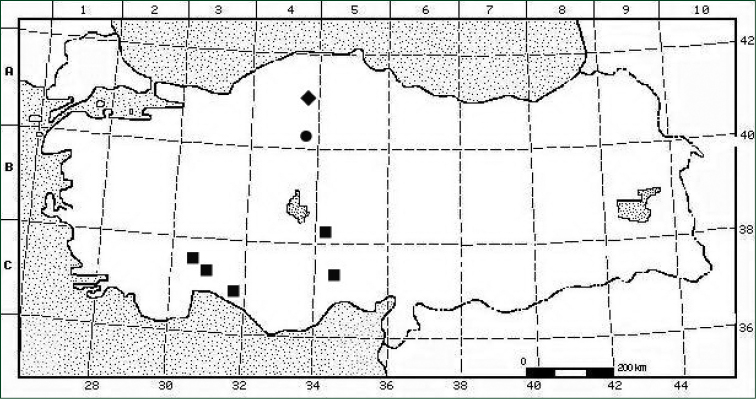
Distribution of Seseli gummiferum subsp. ilgazense (♦), Seseli gummiferum subsp. gummiferum (●) and *Seseli corymbosum* (■) in Turkey.

**Figure 6.**
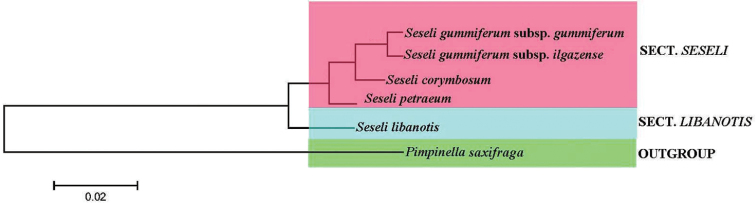
The neighbour joining tree generated using nrITS DNA sequences of some *Seseli* taxa and *Pimpinella saxifraga*.

Figs 1 , 2 , 3 , 4 , 5 , 6

*Affinis Seseli gummiferum Pall. ex Sm. subsp. gummiferum sed umbellis centralibus radiis 13–21 (nec 25–30), bracteis 2–7 (nec plerumque 8–15), umbellis lateralibus radiis 7–13 (nec 15–20), ovariis glabris (nec cum pilis), fructibus glabris (nec cum pilis) differt*.

##### Type.

TURKEY. A4 Kastamonu: Ilgaz Mountain Natural Park, Kastamonu road, from Çatören village to Büyük Hacet Hill, 6 km, in open *Pinus sylvestris* L. and *Abies nordmanniana* (Stev.) Spach. mixed forest, serpentine stony slopes, 41°06'344"N, 33°48'628"E, 1465 m, 22 August 2008, *A.Duran* 8135, *Ö.Çetin & M.Öztürk* (holotype KNYA! isotypes ANK!, GAZI!, HUB!).

##### Description.

Plants monocarpic, 15–30 cm tall; rootstock thickened, cylindrical, oblong, ± vertical, 8–15 mm diameter; stems 3 to 5, terete, finely ridged, mostly glabrous, sparsely puberulent above, with a developed fibrous collar 2.5–7 cm; stems mostly branching from the base, below and rarely at the middle part, green to purplish green. Basal leaves ovate to oblong-ovate, 2-pinnate, 6–20 × 3–10 cm, glabrous; ultimate segments lanceolate, linear-oblong, 5–15 × 0.5–1.5 mm, acute to acuminate; sheaths developed, distinctly widened at base, upper sheath surface sulcate, margin membranous; cauline leaves similar to basal leaves, partly reduced, amplexicaule, middle and upper portions of the stem leafless. Flowers hermaphroditic; the central umbel stout, 7–11 cm diam., equal to or longer than lateral umbels, rarely shorter, with 13 to 21 rays, 1.8–5.5 cm long, puberulent above, unequal, each central umbel with by 2 to 7 bracts; bracts lanceolate, rarely widened at base, 10–17 mm long, margin ± membranous, sometimes bifid to trifid, glabrous or puberulent; umbellules 9–15 mm diam, each comprising 55 to 70 flowers; bracteoles 15–20, connate at base, 5–8 mm long, linear- lanceolate, acuminate, entire or bifid to trifid, green to partly purple, margin distinctly membranous, especially ciliate at apex, out side glabrous to puberulent; lateral umbels 3.5–6 cm diam. with 7 to 13 rays, 1–3.5 cm long, with bracts or without. Flowers subsesile, glabrous, sepals ±purplish, broadly lanceolate, glabrous, persistent, ca. 1 mm; petals white, glabrous with deflexed apex; filament white, anther clearly purple; ovary glabrous. Fruit oblong-elliptic, 3.5–4 × 1.5–2 mm, glabrous, with 5 ribs prominent, obtuse; stylopodium short-conical; styles deflexed in fruit, distinctly purple.

##### Distribution and IUCN red list category.

The new subspecies is known only from the type locality, with specimens collected only from Ilgaz Mountain National Park (Kastamonu province) in Turkey, where the species seems to be very rare. This area is ca. 1.5 km^2^, and mature individuals of the type population number approximately 125. The location is very close to the road side and near forest management. The population is going to be negatively affected from cars, trucks and people in the future. The habitat of this subspecies is clearly under threat of destruction, and therefore, the taxon should be considered Critically Endangered (CR), according to IUCN Red List Criteria (IUCN 2001).

##### Habitat and ecology.

This new subspecies grows at 1450–1470 m with *Lapsana communis* L., *Abies nordmanniana* (Stev.) Spaach, *Centaurea drabifolia* Sm., *Erysimum thyrsoideum* Boiss., *Pteridium aquilinum* (L.) Kuhn, *Sorbus umbellata* (Desf.) Fritsch, *Valeriana alliariifolia* Adams, *Eryngium giganteum* M.Bieb., Bupleurum falcatum L. subsp. persicum (Boiss.) Koso-Pol., *Salvia verticillata* L., *Teucrium chamaedrys* L., *Dactylis glomerata* L., Asyneuma rigidum (Willd.) Grossh. subsp. rigidum.

##### Discussion and conclusion.

Turkey is the most complex country in the Middle East with regard to geographic structure and landforms. It’s comprised of comparatively narrow and long, variously oriented mountain chains, separated by deep valleys and also high- and medium-elevational plateaus. The geological composition and physical direction, exposure and altitude of these mountains are here largely influential not only upon the diversity of vegetation, but also on the richness of the flora (Zohary 1973). Ilgaz Mountain, which is situated in a transitional zone in central and North of Anatolia and is generally composed of serpentine, schist and volcanic rocks. The mountain is orogenically interesting, with the quite active north Anatolian fault found along the southern slopes of Ilgaz Mountain (Kuter 2008). One of the more important reasons for protecting the Ilgaz Mountain is the richness and endemism of its flora. Approximately 100 endemic plants occur within the boundaries of the National Park and the type localities of 19 endemic taxa are found on Ilgaz Mountain. *Delphinium ilgazense* P.H.Davis, *Arabis abietina* Bornmüller, *Draba anatolica* A.Duran & Dinç, *Astragalus nabelekii* Czeczott, *Heracleum paphlagonicum* Czeczott, *Hieracium macrogonum* (Zahn) P.D.Sell & C.West and *Hieracium tuberculatum* Freyn, *Festuca ilgazensis* Markgr.-Dann. are all noteworthy endemic taxa confined to the national park (Davis 1965–1985, Davis et al. 1988, Duman 2000, Duran et al. 2008).

Seseli gummiferum subsp. ilgazense is closely related to two other subspecies found in Turkey. Seseli gummiferum subsp. gummiferum is distributed in Crimea, Central Anatolia (Shishkin 1950, Ball 1968, Duman 2000). The new subspecies differs from Seseli gummiferum subsp. gummiferum, based on its glabrous fruits (not with indument), fewer central umbels, with 13 to 21 rays (not 25 to 30), fewer lateral umbels, with seven to 13 rays (not 15 to 20), as well as the bracteoles 15 to 20 (not 11 to 16) (Hedge and Lamond 1972, Duman 2000).

Seseli gummiferum subsp. ilgazense also differs from Seseli gummiferum subsp. crithmifolium (DC.) P.H.Davis, which is distributed in west and east Crete, Folegandros, Sikinos, Amargos, Karpathos, Saria and neigbouring islands in Aegean Sea. The new taxon is not so broadly distributed, endemic and found only in northern Anatolia (Ilgaz Mountain). Seseli gummiferum subsp. crithmifolium principally differs from subsp. ilgazense by its puberulent fruits, the central umbels with 20–45 rays, and the oblong leaf segments (6–) 10–30 × 2–5 mm (Ball 1968, Hedge and Lamond 1972).

### Key to closely related *Seseli gummiferum* subspecies

**Table:** 

1	Fruit glabrous; the central umbel 13 to 21 rays; lateral umbels with 7 to 13 rays	**subsp. ilgazense**
–	Fruit with hairs; the central umbel 22 to 70 rays; lateral umbels with 13 to 47 rays	
2	Basal leaves glabrous	**subsp. crithmifolium**
–	Basal leaves puberulent or finely pubescent	**subsp. gummiferum**

Since Morison’s (1672), *Plantarum umbelliferarum*, fruit morphology and anatomy have been regarded as essential to the taxonomy of Apiaceae (Drude 1898, Constance 1971, Spalik et al. 2001). Details of the fruits have been traditionally viewed as rich sources of taxonomic characters, exhibiting some, but not excessive variation in features such as fruit shape, the degree and direction of mericarp compression, modifications of the pericarp ribs (e.g. wings or spines), and the shape of mericarp commissural faces. Thus, most traditional classifications of Apiaceae have relied almost exclusively on fruit characters (Plunkett and Downie 1999, Pimenov et al. 2004). Seseli gummiferum subsp. ilgazense has some distinctive characteristics in terms of carpological features. Mericarps of Seseli gummiferum subsp. ilgazense have five large vascular bundles situated beneath the each rib. Large vittae present in mesocarp layer adjacent to the endocarp. There are also two large vittae in the commissure. Differing from Seseli gummiferum subsp. gummiferum, the new subspecies does not have short secretory ducts around vascular bundles. In addition, the vittae around the endocarp are always large and elliptical. The endosperm is round shaped. Commissure width is approximately equal to mericarp width.

Diagnostic characters of Seseli gummiferum subsp. ilgazense with the three related taxa are provided in Table 1.

**Table 1.** Diagnostic characters of Seseli gummiferum subsp. ilgazense, Seseli gummiferum subsp. gummiferum, Seseli gummiferum subsp. crithmifolium and *Seseli corymbosum*.

Characters	subsp. ilgazense	subsp. gummiferum	subsp. crithmifolium	*Seseli corymbosum*
Stems per plant	3 to 5	1 to 4	solitary	solitary
Stem branching	mostly below, rarely in middle portion	below the middle	above the middle	above the middle
Central umbel rays	13 to 21	25 to 30	22 to 45	30 to 70
Lateral umbel rays	7 to 13	15 to 20	17 to 35	13 to 47
Bracts	2 to 7	mostly 8 to 15	mostly absent, rarely up to 2	0 to 1
Bracteoles	15 to 20	11 to 16	19 to 26	19 to 23
Petals	glabrous	glabrous	pubescent	pubescent
Ovary	glabrous	pubescent	puberulent	pubescent
Fruit	glabrous	pubescent	pubescent	pubescent

Five *Seseli* taxa and *Pimpinella* were evaluated in the phylogenetic analysis. PCR amplification with ITS 4/ITS 5 primers generated bands ranging from 595 to 665 bp. Alignment of the ITS sequences was done using Bioedit. Neighbour-Joining (NJ) tree were constructed using Seaview programme (Figure 6). According to NJ analyses, the taxa of *Pimpinella* placed in first clade. This species was used as outgroup. The second clade includes *Seseli libanotis*, which is found in Section *Libanotis*. *Seseli libanotis* is separated from *Seseli gummiferum* and *Seseli petraeum* by having highly wide leaf segment. The second clade incudes *Seseli petraeum*, *Seseli corymbosum*, *Seseli gummiferum* in sect. *Seseli*. *Seseli petraeum* is close to *Seseli gummiferum*. It mainly differs from *Seseli gummiferum* because it has free bracteol from base and main umbel shorter than lateral umbels. *Seseli corymbosum* (Boiss. & Heldr.) P.H.Davis which only occurs in South Anatolia, is readily distinguished from *Seseli gummiferum*, by its solitary stem branching above, bracts either lacking or one below the central umbel, more numerous bracteoles, 19 to 23, larger central umbels with 30 to 70 rays and the lateral umbels with 13 to 47 rays, and finally by the pubescent petals and fruits (Hedge and Lamond 1972). Although Seseli gummiferum subsp. ilgazense is closely related to Seseli gummiferum subsp. gummiferum, this taxa is treated as different subspecies.

## Supplementary Material

XML Treatment for Seseli gummiferum ilgazense

## Appendix

**Representative specimens examined** (*which used for DNA samples): – ***Seseli corymbosum***: Turkey, C3 Antalya: Akseki, Pınarbası village, S of Gidefi Mountain, *A.Duran* 2970 (GAZI); Akseki, Gidefi Mountain, *A.Duran* 1847 (GAZI); C5 Niğde; Ulukışla, between Alihoca-Maden villages, *A.Duran* 6078* (KNYA). – **Seseli gummiferum subsp. gummiferum**: Turkey, A4 Ankara: Hasanoğlan, İdris Mountain, above Adilahmet village, *M.Koyuncu* 16348 (GAZI); Hasanoğlan, İdris Mountain, 1500–1600 m., 14.09.2002, *E.Doğan* 1650* (GAZI). – **Seseli gummiferum subsp. crithmifolium**: Greece, Dodecanese, Karpathos, side of Kali Limni, 10 m, 21.07.1950, *Davis* 18010 (E). – ***Seseli petraeum***: Turkey, Trabzon, between Araklı-Bayburt, 27.07.2002, *A.Duran* 6059* (KNYA). – ***Seseli libanotis***: Turkey, Erzurum, between İspir-Ovit, 1700 m, 18.08.2013, *A.Duran* 9778* (KNYA).
